# Intranasal Chromium Induces Acute Brain and Lung Injuries in Rats: Assessment of Different Potential Hazardous Effects of Environmental and Occupational Exposure to Chromium and Introduction of a Novel Pharmacological and Toxicological Animal Model

**DOI:** 10.1371/journal.pone.0168688

**Published:** 2016-12-20

**Authors:** Abeer Salama, Rehab Hegazy, Azza Hassan

**Affiliations:** 1 Pharmacology Department, Medical Division, National Research Centre, Giza, Egypt; 2 Pathology Department, Faculty of Veterinary Medicine, Cairo University, Giza, Egypt; National Institue on Drug Abuse, UNITED STATES

## Abstract

Chromium (Cr) is used in many industries and it is widely distributed in the environment. Exposure to Cr dust has been reported among workers at these industries. Beside its hazardous effects on the lungs, brain injury could be induced, as the absorption of substances through the nasal membrane has been found to provide them a direct delivery to the brain. We investigated the distribution and the effects of Cr in both brain and lung following the intranasal instillation of potassium dichromate (inPDC) in rats. Simultaneously, we used the common intraperitoneal (ipPDC) rat model of acute Cr-toxicity for comparison. Thirty male Wistar rats were randomly allocated into five groups (n = 6); each received a single dose of saline, ipPDC (15 mg/kg), or inPDC in three dose levels: 0.5, 1, or 2 mg/kg. Locomotor activity was assessed before and 24 h after PDC administration, then, the lungs and brain were collected for biochemical, histopathological, and immunohistochemical investigations. Treatment of rats with ipPDC resulted in a recognition of 36% and 31% of the injected dose of Cr in the brain and lung tissues, respectively. In inPDC-treated rats, targeting the brain by Cr was increased in a dose-dependent manner to reach 46% of the instilled dose in the group treated with the highest dose. Moreover, only this high dose of inPDC resulted in a delivery of a significant concentration of Cr, which represented 42% of the instilled dose, to the lungs. The uppermost alteration in the rats locomotor activity as well as in the brain and lung histopathological features and contents of oxidative stress biomarkers, interleukin-1β (IL-1β), phosphorylated protein kinase B (PKB), and cyclooxygenase 2 (COX-2) were observed in the rats treated with inPDC (2 mg/kg). The findings revealed that these toxic manifestations were directly proportional to the delivered concentration of Cr to the tissue. In conclusion, the study showed that a comparably higher concentrations of Cr and more elevated levels of oxidative stress and inflammatory markers were observed in brain and lung tissues of rats subjected to inPDC in a dose that is just 0.13 that of ipPDC dose commonly used in Cr-induced toxicity studies. Therefore, the study suggests a high risk of brain-targeting injury among individuals environmentally or occupationally exposed to Cr dust, even in low doses, and an additional risk of lung injury with higher Cr concentrations. Moreover, the study introduces inPDC (2 mg/kg)-instillation as a new experimental animal model suitable to study the acute brain and lung toxicities induced by intranasal exposure to Cr compounds.

## Introduction

The wide environmental distribution of chromium (Cr) leads to an increased interest of its toxicity and biological effects. Cr exists mainly in two states, the trivalent (CrIII) and hexavalent (CrVI); both are used in various industrial activities such as steel works, metal finishing, petroleum refining, Cr electroplating, and leather tanning [1, 2].

CrVI is a strong oxidizer and therefore harmful to the biological systems. It is very soluble and thus highly mobile in the environment and presents high toxicity, mutagenic, teratogenic, and carcinogenic effects [3]. It can generate reactive oxygen species (ROS) during their reduction, and these ROS can cause injury to the cellular proteins, lipids, and DNA [4, 5]. Involvement of inflammation in the Cr-toxicity has been also reported [6]. The propagation of this inflammatory response is dependent upon the stimulation of signaling pathways within the cell that include many components work sequentially to control the reaction and to express cytokines [7].

Inhalation of Cr dusts or aerosols by the workers at chromate production and chrome plating has been reported; this has been found to induce perforation of the nasal septum, and respiratory diseases, including hyperplasia of the bronchial epithelium and fibrosis among these workers [8]. Moreover, environmental exposure to urban complex mixtures of particulate matter (PM) containing Cr has been stated as an undisputed risk factor for lung cancer [9, 10]. CrVI has been listed as a hazardous air pollutant (HAP) by the US Environmental Protection Agency (2004), as it is emitted from anthropogenic sources including industries, fuel combustion, and corrosion inhibition [11–13].

Remarkably, intranasal lumen has been found to provide a high absorbance of the substances pass through the intranasal cavity; this is due to the advantages of its rich vasculature, large surface area and highly permeable membrane, together with the avoidance of the first pass metabolism [14, 15]. More importantly, it has been found that a large part of these substances, absorbed nasally, could be delivered directly to the brain within minutes along both the olfactory and trigeminal nerves [16]. In animals, several metals have been shown to pass via the olfactory receptor neurons from the nasal lumen through the cribriform plate to the olfactory bulb. Some metals can cross synapses in the olfactory bulb and migrate via secondary olfactory neurons to the distant nuclei of the brain [17]. However, the absorbance of Cr through the intranasal membrane and its delivery and effect in the brain was not investigated before.

Therefore, the aim of this study was to investigate the delivery of Cr to both brain and lung tissues following an intranasal (i.n.) administration of potassium dichromate (PDC) in rats. In addition, the study of the changes in the locomotor activity, oxidative stress biomarkers levels in those tissues, and the expression of interleukin-1β (IL-1β), phosphorylated protein kinase B (PKB), and cyclooxygenase 2 (COX-2), as signaling-controllers of the inflammatory response pathways, was considered. Also, we intended to compare the results obtained with those caused by using the common intraperitoneal (i.p.) rat model of acute Cr-toxicity [18].

## Materials and Methods

### Animals

Adult male Albino Wistar rats, weighing 120-140g, were obtained from the animal house colony of the National Research Centre (NRC, Egypt). The animals were maintained at a controlled temperature of 24 ± 1°C with a 12–12 h light-dark cycle (light cycle, 07:00–19:00), and were allowed free access to water and standard chow *ad libitum*. They were treated according to the national and international ethics guidelines stated by the ethics committee of NRC. As well, this study has been approved by the ethics committee of NRC, and all procedures and experiments were performed according to a protocol approved by it. The earliest scientifically justified endpoint was used in this study to prevent pain or distress in the experimental animals, in which the animals were sacrificed by decapitation, under ether anesthesia, for samples collection.

### Chemicals

PDC was purchased from Sigma Aldrich Chemical Co. (USA).

### Experimental design

Thirty rats were randomly allocated into five groups (n = 6). The 1^st^ group received i.n. saline and served as the normal group, while the 2^nd^ group received a single i.p. injection of PDC (ipPDC, 15 mg/kg) and served as the standard model of acute Cr-toxicity group [18]. In the other 3 groups, the rats received PDC intranasally (inPDC) as a single dose of 0.5, 1, or 2 mg/kg, respectively.

### Measurement of the motor activity of the rats

Activity of the rats was measured by detecting their movements using the grid floor activity cage (Model no. 7430, Ugo-Basile, Italy). Rats were acclimatized for 1 hour to the test room before placing the animal in the activity cage [19]. The activity counts of rats were pretested in three successive sessions, each of 5 min duration, before starting the experiment to habituate the animals to the apparatus [20]. Then, the rats were placed in the activity cage and the activity counts were calculated over 5 min durations (before and 24 h following PDC administration).

### Assessment of chromium residues in the brain and lung tissues

Directly after measurement of the rats' activity, the animals were sacrificed by decapitation, under ether anesthesia, for samples collection. No animal died prior to this experimental endpoint. The brain and lungs from each rat were immediately dissected out, rinsed with phosphate-buffered saline (PBS) to remove the excess blood. Weighed parts from all regions of the brain, including frontal, parietal, occipital, and temporal lobes, containing the hippocampus, and olfactory bulb, as well as the cerebellum, and brainstem were homogenized, and also parts from all lobes of the lungs. The content of Cr was determined in brain and lung tissue homogenates by atomic absorption spectrometry (AAS, unicam 969) according to the method described previously [21].

### Brain and lung tissues biochemical analysis

Reduced glutathione (GSH), catalase, lipid peroxides measured as malondialdehyde (MDA), IL-1β, and phosphorylated PKB contents were assessed in the lung and brain tissue homogenates of all animals using specific diagnostic kits: Biodiagnostic (Egypt), komabiotech ELISA kits (Korea), and Cusabio ELISA Kits (Austria)

### Histopathological examination

The different brain and lung lobes tissues from each animal were fixed in 10% neutral buffered formalin, then washed, dehydrated, and embedded in paraffin blocks. Sections of 5 μm thick were stained with haematoxylin and eosin (H&E) [22], for histopathological examination. Five brain and lung sections per group were examined. Ten random high microscopic fields (X40) per section were scanned for assessment of the histopathological lesions using binocular Olympus CX31 microscope. In brain tissues, the main histological parameters used in the evaluation were neuronal degeneration with neuronophagia, satellatosis, and cerebral cortical hemorrhage. For lungs, the main histological parameters used were mucoid degeneration, epithelial desquamation, hyperplasia of the bronchial and bronchiolar epithelium, peribronchial, peribronchiolar and interstitial inflammatory cell infiltrates, foreign body granuloma, thickening of alveolar wall and alveolar edema, as well as activation of mast cells. A semi quantitative lesion-score, scaled from 0 to 4, was used to assess these histopathological alterations according to the method illustrated by Toya *et al* [23] with some modifications. In this grading score system, (0) indicates that the tissue is appeared normal, (1) indicates very slight alterations with sporadic lesions in limited areas, (2) indicates slight alterations with obvious changes demonstrated in limited areas, (3) indicates moderate alterations with lesions confined to one third of the total area of the lobe, and (4) indicates marked alterations with extensive lesions over an area greater than one third of the lobe.

### Immunohistochemical examination

For demonstration of COX-2 enzyme expression in the brain and lung tissues, the tissue sections were deparaffinized and incubated in 3% H_2_O_2_. Blocking of non-specific binding was performed using goat serum. Then, the tissue sections were incubated with a human monoclonal anti-COX-2 (Cayman Chemical, Ann Arbor, MI, USA). The immune reaction was visualized using diaminobenzidene (DAB, Sigma Chemical Co, USA). The positive COX-2 immune reactive cells were counted in three random high microscopic fields X40.

### Statistical analysis

All the values are presented as means ± standard error of the means (SE). Comparisons between different groups were carried out using one-way analysis of variance (ANOVA) followed by Tukey HSD test for multiple comparisons. Graphpad Prism software, version 5 (Inc., USA) was used to carry out these statistical tests. The difference was considered significant when *p* < 0.05.

## Results

### Effect of potassium dichromate on motor activity

**Fig 1:**
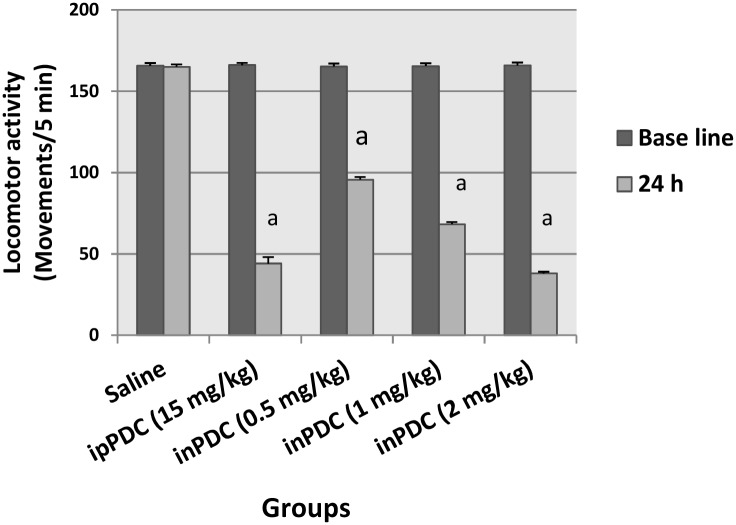
Effect of potassium dichromate on motor activity. Saline, rats received intranasal instillition of saline; inPDC, rats received intranasal instillition of potassium dichromate; ipPDC, rats received intraperitoneal injection of potassium dichromate. ^a^ significantly different from the basal value of the same group at *p*<0.05.

Treatment of rats with inPDC (0.5, 1 or 2 mg/kg) decreased their motor activity after 24 h by 42%, 59% and 77% compared to their basal values. In comparison to the ipPDC-treated group, the high dose of inPDC induced a 14% more decrease in the rats' motor activity (Fig 1).

### Chromium concentration in brain and lung tissues

**Fig 2:**
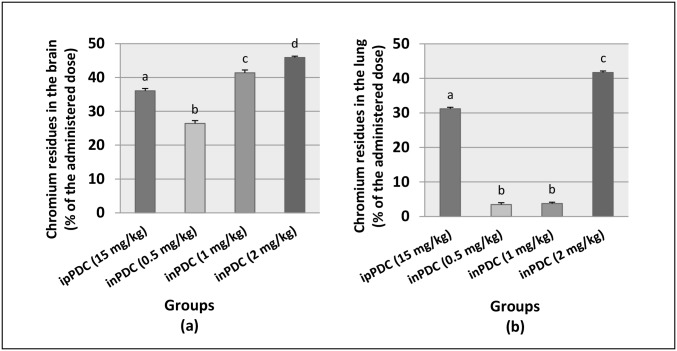
Chromium residues in the brain (a) and lung (b) tissues. Saline, rats received intranasal instillition of saline; inPDC, rats received intranasal instillition of potassium dichromate; ipPDC, rats received intraperitoneal injection of potassium dichromate. Groups with different letters are significantly different at *p*<0.05.

Treatment of rats with ipPDC resulted in a delivery of 36% and 31% of the injected dose of Cr to the brain and lung tissues, respectively. In rats treated with inPDC, the delivery of Cr to the brain was increased in a dose-dependent manner, as the percentages detected were 26%, 41% and 46% of the inPDC (0.5, 1 or 2 mg/kg) contents of Cr, respectively. On the other hand, non-significant traces of Cr residues were detected in the lung tissues following instillation of 0.5 or 1 mg/kg inPDC. However, 42% of the instilled Cr was detected in the lungs of rats treated with inPDC 2 mg/kg (Fig 2).

### Brain tissues biochemical analysis

Treatment of rats with inPDC (0.5, 1, 2 mg/kg) increased the brain MDA up to 1.21-fold, 1.61-fold, and 2.21-fold the normal value, respectively. On the other hand, no marked effect was produced by the lowest dose of inPDC on the brain contents of GSH and catalase. However, inPDC (1 mg/kg) resulted in a significant decrease of the normal concentrations of GSH and catalase in the brain to 88% and 72%, respectively, and 75% and 30% of the normal values, respectively, were observed in the brains of rats treated with inPDC (2 mg/kg).

**Fig 3:**
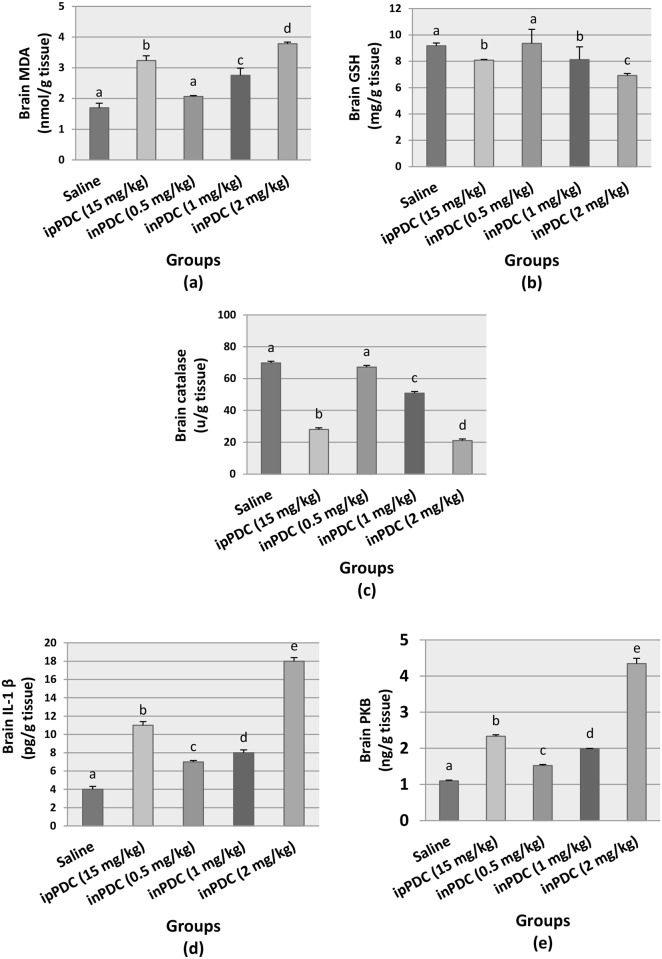
Effect of potassium dichromate on brain contents of (a) MDA, (b) GSH, (c) catalase, (d) IL-1β, and (e) PKB. Saline, rats received intranasal instillition of saline; inPDC, rats received intranasal instillition of potassium dichromate; ipPDC, rats received intraperitoneal injection of potassium dichromate. Groups with different letters are significantly different at *p*<0.05.

Compared to ipPDC-injected rats, brain MDA, GSH, and catalase contents in rats treated with the highest dose of the inPDC were 117%, 85%, and 75% of the observed values, respectively (Fig 3a, 3b and 3c).

The normal brain content of IL-1β in inPDC (0.5, 1, 2 mg/kg) was significantly increased to 1.56-fold, 1.96-fold and 4.07-fold, respectively. Also, phosphorylated PKB content was significantly increased to 1.39-fold, 1.82-fold and 3.98-fold, respectively, compared to normal brain contents. Brain IL-1β and PKB contents in rats treated with the highest dose of the inPDC were 1.64-folds and 1.85-folds, respectively, those observed in ipPDC-injected rats (Fig 3c and 3d).

### Lung tissues biochemical analysis

**Fig 4:**
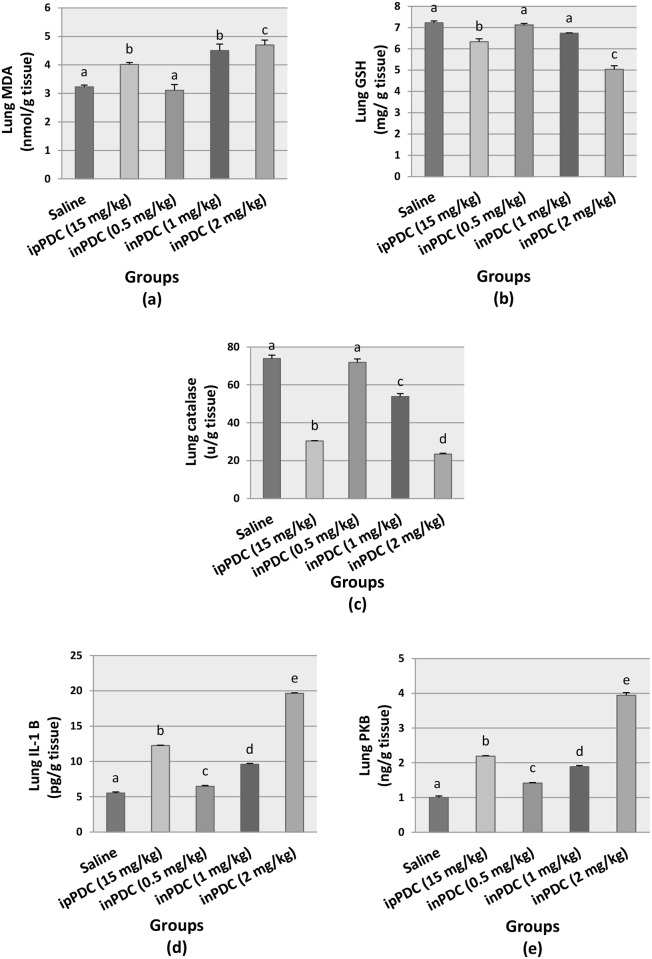
Effect of potassium dichromate on brain contents of (a) MDA, (b) GSH, (c) catalase, (d) IL-1β, and (e) PKB. Saline, rats received intranasal instillition of saline; inPDC, rats received intranasal instillition of potassium dichromate; ipPDC, rats received intraperitoneal injection of potassium dichromate. Groups with different letters are significantly different at *p*<0.05.

A significant increase in the lung content of MDA was observed in rats treated with inPDC as compared to the normal group. The lung content of MDA in inPDC (1, 2 mg/kg)-treated groups were significantly increased to 1.39-fold and 1.45-fold, respectively; while a concentration of lung GSH in inPDC-treated group (2 mg/kg) was significantly decreased to about 71% of the normal value. In addition, the normal lung content of catalase in inPDC-treated group (1, 2 mg/kg) were significantly decreased to 72% and 31%, respectively. Rats treated with the highest dose of inPDC showed MDA, GSH, and catalase contents in the lung tissues that were 117%, 79%, and 77%, respectively, those observed in ipPDC-injected rats (Fig 4a, 4b and 4c).

Lung IL-1β contents in inPDC (0.5, 1, 2 mg/kg) were significantly elevated to 1.17-fold, 1.73-fold and 3.55-fold the normal levels, respectively. In addition, phosphorylated PKB content was significantly increased to 1.41-fold, 1.88-fold and 3.94-fold, respectively. Lung IL-1β and PKB contents in rats treated with the highest dose of the inPDC were increased to 1.60-folds and 1.80-folds, respectively, compared to those observed in ipPDC-injected rats (Fig 4c and 4d).

### Histopathological examination findings

Tables 1 and 2 summarize the histopathological alterations demonstrated in the brain and lung of normal and treated rats.

**Table 1:** The main histopathological alterations demonstrated in brain tissues.

Parameters	Groups				
	Saline	ipPDC (15 mg/kg)	inPDC (0.5 mg/kg)	inPDC (1 mg/kg)	inPDC (2 mg/kg)
**Cerebral cortical hemorrhage**	_	Multifocal and confined to the white matter	Focal hemorrhage in grey and white matter	Multifocal hemorrhage in grey and white matter	Extensive hemorrhage in grey and white matter and in the perivascular space
**Neuronal degeneration**	Confined to sparse neuronal cell	Confined to individual neuronal cells in all sections	Affecting Large number of neuronal cells	Wide spread neuronal degeneration	Wide spread neuronal degeneration
**Satellatosis**	Few	Mild	Mild	Diffuse	Diffuse

Saline, rats received intranasal instillition of saline; inPDC, rats received intranasal instillition of potassium dichromate; ipPDC, rats received intraperitoneal injection of potassium dichromate.

**Table 2:** The main histopathological alterations demonstrated in the lungs.

Sites/Lesions		Groups				
		Saline	ipPDC (15 mg/kg)	inPDC (0.5 mg/kg)	inPDC (1 mg/kg)	inPDC (2 mg/kg)
**Bronchi**	**Epithelial desquamation**	0	3	3	4	4
	**Epithelial hyperplasia**	1	4	3	4	4
	**Peribronchial inflammatory cell infiltration**	1	3	2	3	4
**Bronchioles and interstitial**	**Epithelial desquamation**	1	3	2	2	4
	**Epithelial hyperplasia**	2	4	3	4	4
	**Granuloma**	0	0	2	4	4
	**Peribronchiolr and interstitial inflammatory cell infiltration hyperplasia**	1	4	3	4	4
**Alveoli**	**Increase foamy macrophages**	0	2	3	3	4
	**Thickening of alveolar wall**	1	4	3	4	4
	**Alveolar edema**	0	1	1	4	4
**Mucosal mast cell activation**		0	2	1	3	4

0 = negative; 1 = very slight; 2 = slight; 3 = moderate; 4 = severe.

Saline, rats received intranasal instillition of saline; inPDC, rats received intranasal instillition of potassium dichromate; ipPDC, rats received intraperitoneal injection of potassium dichromate.

**Fig 5:**
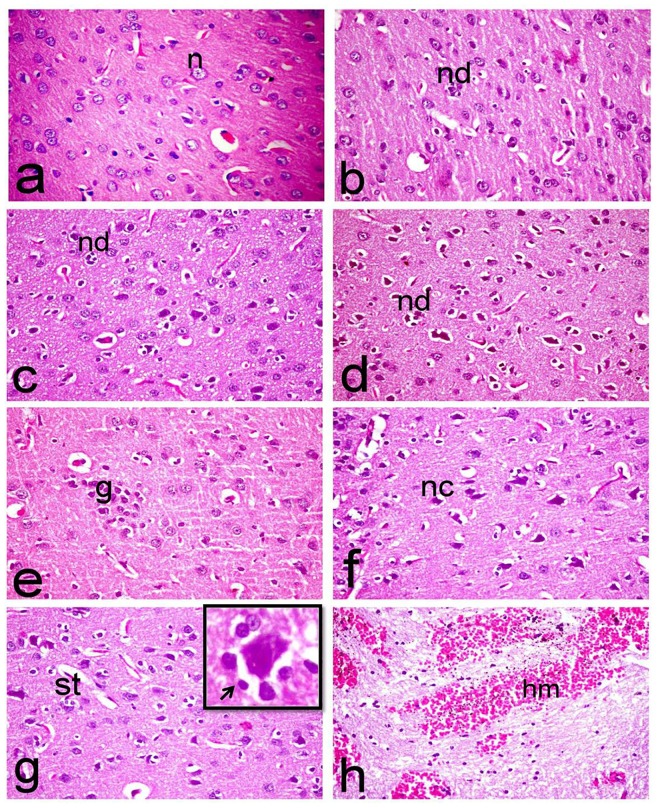
Histopathological investigation of brain tissues. Brain sections of **(a) normal rats** showing normal morphology of cerebral cortex with normal rounded neuronal cells *(n)*, **(b) ipPDC-treated rats** showing gliosis and degeneration of individual neuronal cells associated with neuronophagia, **(c) inPDC (0.5 mg/kg)-treated rats** showing wide spread neuronal cell degeneration *(nd)* with neuronophagia of the degenerated neurons as well as vacuolation of the neuropil, **(d & e) inPDC (1 mg/kg)-treated rats** showing (d) neuronal cell degeneration *(nd)* associated with neuronophagia and neuronal loss, (e) focal gliosis *(g)*, and **(f, g & h) inPDC (2 mg/kg)-treated rats** showing (f) neuronal cell necrosis *(nc)* with intensely eosinophilic shrunken neuronal cell bodies, (g) proliferation of glia cells, satellatosis *(st)*, and neuronophagia (insert) in which the degenerated neuron is surrounded by astrocytes and microglia cells (arrow) (h) extensive hemorrhage *(hm)* in the white matter. (H&E, X40).

Brain sections of normal rats showed normal morphology of cerebral cortex with normal rounded neuronal cells (Fig 5a). Whereas, brain of ipPDC-treated rats showed degeneration of individual neuronal cells associated with neuronophagia, satellatosis, and gliosis (Fig 5b). Mild proliferation of glia cells and focal small hemorrhage in the white matter were also demonstrated. Brain of inPDC (0.5 mg/kg)-treated rats showed wide spread neuronal cell degeneration with neuronophagia of the degenerated neurons as well as vacuolation of the neuropil (Fig 5c). Satellatosis, multifocal gliosis and extensive hemorrhage of the white matter were characteristic alterations demonstrated in this group. Similar histopathological alterations were demonstrated in inPDC (1 mg/kg)-treated rats, as neuronal cell degeneration associated with neuronophagia and neuronal loss were frequently demonstrated (Fig 5d). Focal gliosis (Fig 5e), meningeal and extensive cerebral cortical hemorrhage were also significant. Brain of inPDC (2 mg/kg)-treated rats revealed the most severe histopathological lesions, compared to other treated groups. These lesions were manifested by neuronal cell necrosis with intensely eosinophilic shrunken neuronal cell bodies (Fig 5f) associated with proliferation of glia cells and neuronophagia of the degenerated neurons (Fig 5g), in addition to extensive neningeal, perivascular and cerebral cortical hemorrhage which was characteristically demonstrated in the white matter (Fig 5h).

**Fig 6:**
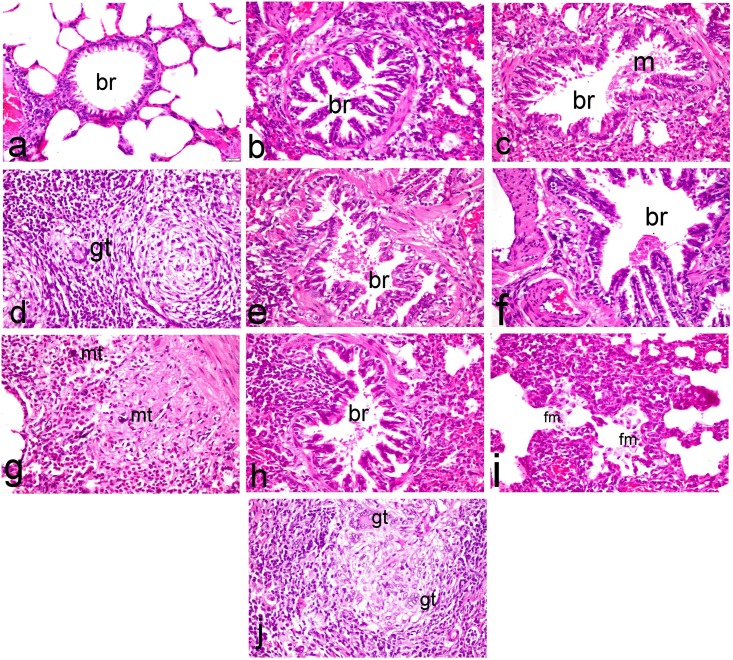
Histopathological investigation of lung tissues. Lung sections of **(a) normal rats** showing normal pulmonary parenchyma with normal bronchiolar epithelium *(br)* and normal alveoli, **(b) ipPDC-treated rats** showing hyperplasia of the bronchiolar epithelium with peribronchiolar leukocytic cell infiltration, **(c & d) inPDC (0.5 mg/kg)-treated rats** showing (c) proliferation of bronchiolar epithelium associated with apoptotic changes and intraluminal aggregation of mucous materials *(m)* mixed with desquamated epithelium, (d) non-caseating granuloma with presence of giant cells *(gt)*, **(e) inPDC (1 mg/kg)-treated rats** showing epithelial shedding and intraluminal aggregation of mucous, and **(f, g, h, i & j) inPDC (2 mg/kg)-treated rats** showing (f) papillary hyperplasia of bronchial epithelium, (g) intense infiltration of bronchial mucosa and bronchial wall with inflammatory cells concurrently with activation of mast cells *(mt)*, (h) hyperplasia of the epithelial lining terminal bronchioles with intense peribronchilar leukocytic cell infiltration, (i) marked thickening of the alveolar wall with intraluminal aggregation of foamy macrophages *(fm)*, (j) granuloma with aggregation of multiple giant cells of foreign type. (H&E, X40).

Lungs of normal rats showed normal pulmonary parenchyma with normal bronchiolar epithelium and normal alveoli (Fig 6a). Meanwhile, lung of ipPDC (15 mg/kg)-treated rats revealed hyperplasia of the bronchiolar epithelium with peribronchiolar leukocytic cell infiltration (Fig 6b). Thickening of pulmonary parenchyma with proliferating macrophages and infiltrating mononuclear cells as well as congestion of interstitial blood capillaries were demonstrated in this group. Similar histopathological alterations were demonstrated in inPDC (0.5 mg/kg)-treated rats represented by hyperplastic proliferation of bronchiolar epithelium associated with apoptotic changes and intraluminal aggregation of mucous materials mixed with desquamated epithelium, in addition to infiltration of the peribronchiolar tissue with inflammatory cells (Fig 6c). The pulmonary parenchyma revealed intense focal interstitial inflammatory cell infiltrates with marked thickening of the alveolar wall with presence of emphysematous alveoli. One of the most characteristic lesion demonstrated in this group was presence of multiple non-caseating granulome embeded in the pulmonary parenchyma. Each granulome is consisted of intense focal aggregation of epithelioid cells, macrophages, lymphocytes and giant cells (Fig 6d). On the other hand, lung of inPDC (1 mg/kg)-treated rats showed papillary hyperplasia of large bronchial epithelium and or goblet cells associated with hyperplasia of the peribronchial lymphoid tissue. The bronchioles showed epithelial shedding, hyperplasia of the bronchiolar epithelium and intraluminal aggregation of mucous with infiltration of the peribronchiolar tissue with inflammatory cells (Fig 6e). Congestion of the blood vessels with extensive perivascular edema associated with hemorrhage and infiltration of inflammatory cells were also demonstrated. The most severe histopathological lesions were demonstrated in inPDC (2 mg/kg)-treated rats as manifested by hyperplasia of epithelial lining airways and large bronchi (Fig 6f) with intense infiltration of bronchial mucosa and bronchial wall with inflammatory cells. These findings were confirmed in immunohistochemical stained sections, concurrently with activation of mast cells in the peribronchial and perivascular tissue, which are significantly demonstrated in this group (Fig 6g) in addition to hyperplasia of the peribronchial lymphoid tissue. The terminal bronchioles revealed hyperplasia of the epithelial lining with intense peribronchilar leukocytic cell infiltration (Fig 6h). The pulmonary parenchyma revealed marked thickening of the alveolar wall by proliferating macrophages and infiltrating inflammatory mononuclear cells with intraluminal aggregation of foamy macrophages (Fig 6i) in addition to presence of non caseating granulome with aggregation of multiple giant cells of foreign type (Fig 6j).

### Immunohistochemical evaluation of COX-2 enzyme expression

Table 3 illustrates the results of immunohistochemical evaluation of COX-2 immune-stained cells in the brain and lung of normal and treated rats.

**Table 3:** Immunohistochemical evaluation of COX-2 immune-stained cells in the lung and brain tissues of normal and treated rats.

Groups	COX-2 immune-stained cells (count/high microscopic field)	
	In brain tissues	In lung tissues
**Saline**	1.00 ^a^ ±0.57	0.33 ^a^ ±0.33
**ipPDC (15 mg/kg)**	13.33 ^ab^ ±2.60	24.00 ^b^ ±4.58
**inPDC (0.5 mg/kg)**	19.33 ^bc^ ±1.20	22.00 ^b^ ±5.85
**inPDC (1 mg/kg)**	31.00 ^cd^ ±3.46	37.33 ^b^ ±1.85
**inPDC (2 mg/kg)**	40.33 ^d^ ±8.25	90.66 ^c^ ±12.44

Saline, rats received intranasal instillition of saline; inPDC, rats received intranasal instillition of potassium dichromate; ipPDC, rats received intraperitoneal injection of potassium dichromate.

Data are expressed as mean±SE. (*n* = 6).

Different superscripts within the same column are significantly different at *p*<0.05.

**Fig 7:**
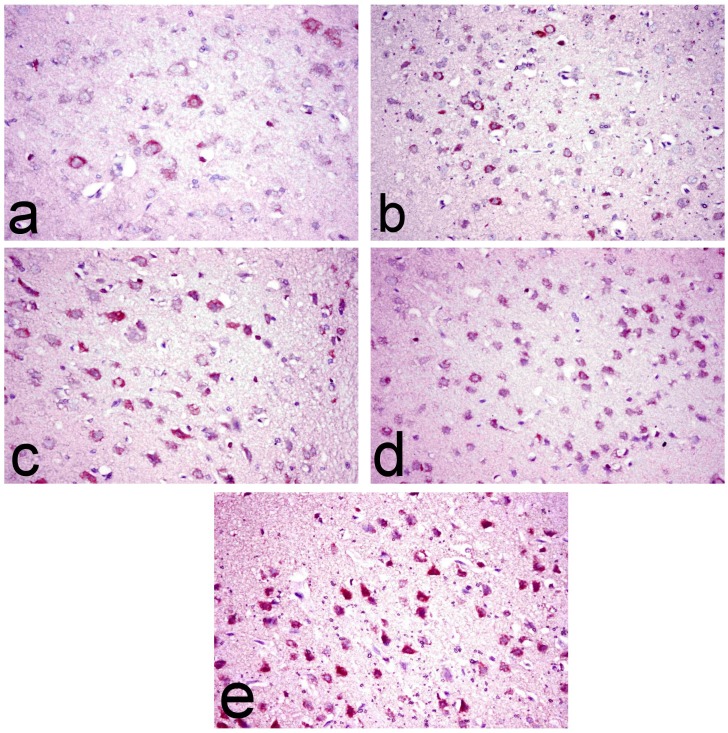
Immunohistochemical investigation of brain tissues. Brain sections of **(a) normal rats** showing scattered individual COX-2 immune-stained cells, **(b) ipPDC-treated rats** showing an increase of COX-2 immune-stained cells with perinuclear immunereactivity, **(c) inPDC (0.5 mg/kg)-treated rats** showing COX-2 immune-stained cells with perinuclear immunereactivity, **(d) inPDC (1 mg/kg)-treated rats** showing significant increase of COX-2 immune-stained cells, and **(e) inPDC (2 mg/kg)-treated rats** showing numerous intensely brown COX-2 immune-stained cells distributed all over the cerebral cortical layer. (COX-2 immunohistochemical staining, X40).

Brain sections of normal rats showed scattered individual COX-2 immune-stained cells (Fig 7a). Whereas, brain of ipPDC (15 mg/kg) and inPDC (0.5 mg/kg)-treated rats revealed significant increase of COX-2 immune-stained cells with perinuclear immunereactivity (Fig 7b and 7c) (13.33±2.60 and 19.33±1.20, respectively) compared to the normal group (1.00±0.57). On the other hand, these COX-2 immune-stained cells were more significantly increased in inPDC (1 mg/kg)-treated group (31.00±3.46) (Fig 7d) compared to the normal and ipPDC groups. The most marked increase in COX-2 immune-stained cells was demonstrated in inPDC (2 mg/kg)-treated rats (40.33±8.25), that appeared numerous and intensely brown and distributed all over the cerebral cortical layer (Fig 7e).

**Fig 8:**
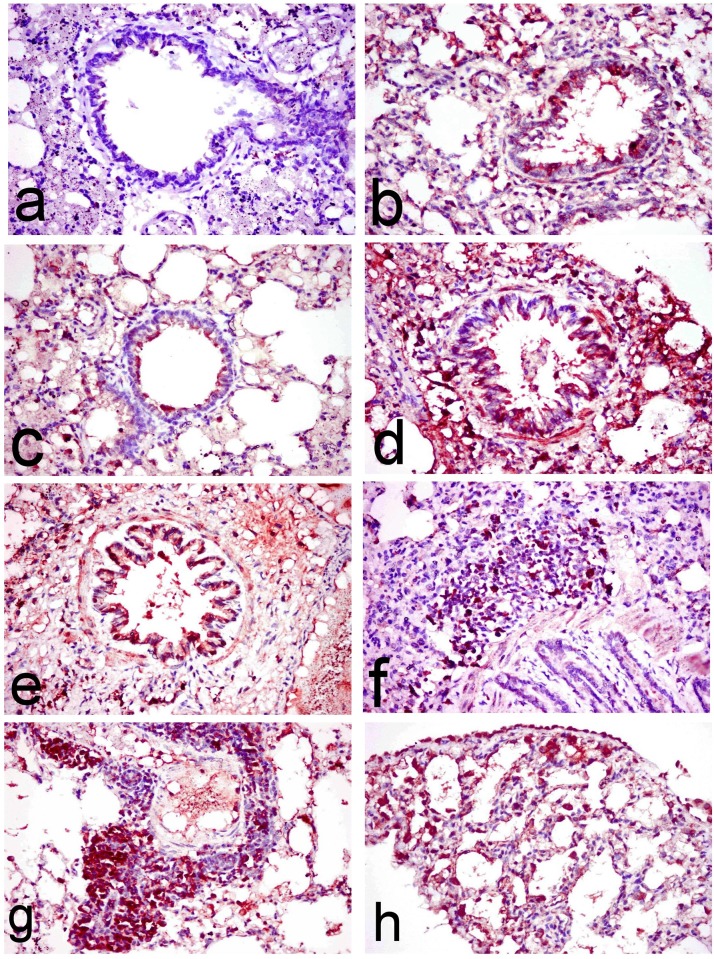
Immunohistochemical investigation of lung tissues. Lung sections of **(a) normal rats** showing no COX-2 immune-stained cells, **(b) ipPDC-treated rats** showing COX-2 immune stained cells lining the bronchiolar mucosa and alveolar wall, **(c) inPDC (0.5 mg/kg)-treated rats** showing COX-2 immune-stained cells lining the bronchiolar mucosa and alveolar wall as well as in the alveolar lumina, **(d) inPDC (1 mg/kg)-treated rats** showing abundant COX-2 immune-stained cells lining the bronchiolar wall and the peribronchiolar tissue, and **(e, f, g & h) inPDC (2 mg/kg)-treated rats** showing (e) COX-2 immune-stained cells that appeared intensely brown lining the bronchiolar wall, (f) COX-2 immune-stained cells in the peribronchial tissue, (g) COX-2 immune-stained cells in the perivascular tissue, and (h) COX-2 immune-stained cells in the alveolar lumen. (COX-2 immunohistochemical staining, X40).

Lungs of normal rats showed no COX-2 immune-stained cells (Fig 8a) except for spars positive cell in some examined sections (0.33±0.33). However, lungs of ipPDC and inPDC (0.5 mg/kg)-treated rats revealed significant increase of COX-2 immune-stained cells lining the bronchiolar mucosa and alveolar wall as well as in the alveolar lumina (24.00±4.58 and 22.00±5.85, respectively) (Fig 8b and 8c). Also, lungs of inPDC (1 mg/kg)-treated rats showed abundant COX-2 immune-stained cells (37.33±1.85) lining the bronchiolar wall, the peribronchiolar tissue and alveolar wall (Fig 8d). On the other hand, a more significant and diffuse increase of COX-2 immune-stained cells were recorded in the inPDC (2mg/kg)-treated rats (90.66±12.44), compared to other treated groups. These COX-2 immune-stained cells appeared intensely brown lining the bronchiolar wall (Fig 8e) and the peribronchial tissue (Fig 8f), in addition to the perivascular tissue (Fig 8g), and intra alveolar lumen (Fig 8h).

## Discussion

The goal of this study was to use a rat model to evaluate the potential brain and lung injuries among individuals environmentally or occupationally exposed to Cr dust. However, in the study, intranasal instillation of PDC was used as an alternative to inhalation of Cr-dust; this is to avoid the downsides of inhalation, and to provide a more accurate control of the exposure concentration. Other research group used the intratracheal rout instead of inhalation for the same reasons [24]. However, in our study we used the intranasal instillation rather than intratracheal to assess, precisely, the effect of passing Cr through the nasal cavity, and to explore its potential absorption through the intranasal mucosa.

In the workers at Cr-based industries, signs and symptoms of adverse nasal and respiratory effects were observed and reported at chronic occupational exposure levels of 0.002–0.2mg CrVI/m^3^ (25). However, the mean level in the breathing zone above the plating baths at electroplating facilities, for example, was found to be 0.414 mg CrVI/m^3^ (26); this is about 20-folds the dose at which the hazardous effects of Cr start to be recognized.

At complete rest, a 70 kg person breathes at a rate of about 0.007 m^3^/min. This rate increases significantly with the activity level to be around 0.012 m^3^/min during light activities, and 0.04–0.06 m^3^/min during moderate exercise (27). Considering the workers as moderately active individuals, their average exposure to Cr could be estimated as 0.005 mg/min. With an average of eight working-hours a day, the average daily occupational exposure dose of Cr is expected to be 2.4 mg. According to Paget and Barnes (28), the equivalent rat-dose is 0.043 mg Cr VI/kg/day, which presents in 0.12 mg of PDC. To investigate the acute effect, the present study considered the single exposure to higher concentrations; this could be due to greater levels of Cr residues in the surroundings, longer duration of contact, and/or faster breathing rates. Therefore, 0.5, 1, and 2 mg/kg of inPDC were used.

Intranasal instillation of PDC produced a dose-dependent increase in Cr distribution to the brain, and this dissemination reach up to 46% of the administered dose in the brains of inPDC (2 mg/kg)-treated rats. This finding suggests that a large portion of the instilled dose is absorbed through the nasal cavity and directly delivered to the brain. In correspondence, many studies showed that absorption of substances through intranasal cavity provides them a direct delivery to the brain through both the olfactory and trigeminal nerves [16]. In agreement, other research groups reported a brain-targeted distribution of manganese [25] and silver [26] following intranasal instillation.

Our finding also showed that other percentage of Cr, especially in highest dose of inPDC, was detected in the lung. This could be explained by the transportation of Cr through the airways to the lungs. This observation is consistent with the detection of high Cr concentrations in the respiratory system of workers in the regions of the chromate industry, and this supports that the intranasal delivery method chosen in this study is effective for modeling human exposure [27].

However, the detection of very low levels of Cr in the lung tissues following the instillation of the low and medium doses of PDC could be due to these doses were not large enough to deliver to the lung and it might affect the upper respiratory tract (need to be investigated).

Interestingly, although we used high dose of ipPDC (7.5 times the highest dose of inPDC), its delivery to the brain and lungs was markedly less. These results indicated that to study the Cr toxicity induced by the exposure to its vapor, ipPDC model is not the proper one to be used; however, inPDC model could simulate the actual event.

The high dose of inPDC, in this work, significantly decreased the motor activity of rats after 24 h; this effect was more remarkable than that caused by ipPDC. Motor activity is used as a parameter to assess the behavioral activity level of the rats. It is a useful tool for assessing locomotive impairment in animal models of neuronal dysfunction and lack of neuromuscular coordination [28]. Therefore, the current decrease in motor activity of the rats pointed out the degree of neurotoxicity and neuronal dysfunction induced by PDC. Correspondingly, the neurotoxic effect of ipPDC and its impact on the motor activity has been reported also in a previous study [29]. Obviously, the locomotor activity of the rats in the present study was the least in the group treated with the highest dose of inPDC harmoniously with the observed delivery of the largest concentration of Cr to the brain following that dose.

Our results indicated that brain and lung oxidative stress was induced in rats by a single PDC administration as evidenced clearly by the increase in MDA and decrease in GSH and catalase contents when compared to the normal brain and lung contents. These findings also showed that the effect of the highest dose of inPDC was more destructive than that brought either by the other doses of inPDC or by ipPDC. The induction of oxidative stress by CrVI has been shown in many studies [4–6, 30]. Moreover, Wang *et al*. reported the oxidative stress and lung injury induced by single intratracheal instillation of PDC [31].

The present work also demonstrated that PDC caused an immediate inflammatory reaction in brain and lungs tissues as evidenced by the upregulation of IL-1β, phosphorylated PKB, and COX-2 in brain and lung 24 h following a single exposure. The study showed that a single dose of inPDC (2 mg/kg) resulted in an acute inflammatory response in brain and lung tissue more than ipPDC (15 mg/kg). Similarly, the neuropathological findings based on comparisons of brain samples collected from accident victims indicate that the brain responds to different types of inhaled air pollution, such as metals, and urban PM with a common pathway of neuroinflammation [32, 33]. Elevated levels of brain IL-1β [33], especially with high exposure concentrations, COX-2 [34], and several protein kinases have been reported and suggested to play major roles [35].

IL-1β is a pluripotent proinflammatory cytokine that is an activator of host defense responses to injury in both the periphery and the central nervous system [36]. In the brain [37] and lungs [38], IL-1β has been found to exacerbate damage resulting from acute insults and induce COX-2 protein expression and prostaglandin E2 production. In line with these previous findings, the present immunohistochemical investigation of brain and lung tissues demonstrated an increase in COX-2 immune stained cells parallel to the increased IL-1β levels.

PKB or Akt is a serine/threonine kinase that is recruited to the plasma membrane in cells stimulated with a variety of stimulants including growth factors and cytokines. Recruitment of PKB to the membrane requires association of its protein domain (the PH domain) with phosphatidylinositol 3,4,5-trisphosphate (PIP_3_), a product of the enzyme phosphoinositide 3-kinase (PI3K). This results in the phosphorylation and activation of PKB [39]. Activated PKB mediates downstream responses, including cell survival, growth, proliferation, cell migration and angiogenesis, by phosphorylating a range of intracellular proteins [40]. Choi *et al*. reported that inflammation induces PKB phosphorylation [41]. Interestingly, PKB appeared to act as a positive as well a negative regulator of inflammatory cytokine production, depending on the nature of the stimulus. However, Rajaram *et al*. found that PKB promotes proinflammatory cytokine production [42] and subsequently exaggerates the inflammatory reaction. Furthermore, other research group reported a potent anti-inflammatory mechanism associated with direct suppression of PKB phosphorylation [43]. In addition, Hao *et al*. [44] explained brain injury mechanism via the activation of PKB. Accordingly, the increased activity of PKB in brain and lung tissues of PDC-treated rats indicated the involvement of PI3K/PKB pathway in the damage induced by Cr in both organs.

The histopathological results in the current study supported the all other findings as they depicted the most severe histopathological lesions in the brain and lung tissues of rats treated with inPDC (2 mg/kg) in the form of acute injury, inflammatory reaction and apoptotic changes. The severe brain lesions that were demonstrated in inPDC groups denoting the effective penetration of Cr across the nasal mucosa and induction of brain toxicity. Moreover, the revealed foreign body granuloma that were only demonstrated in inPDC (2 mg/kg) are in complete agreement with Toya et al. [23] who demonstrated alveolar and interstitial inflammation and granuloma formation in rats upon intratracheal instillation of chromate compound. These granuloma could be explained by the irritation induced by chromium which is well known as respiratory and mucous irritant [45].

## Conclusion

In conclusion, the study showed that a comparably higher concentrations of Cr and more elevated levels of oxidative stress and inflammatory markers were observed in brain and lung tissues of rats subjected to inPDC in a dose that is just 0.13 that of ipPDC dose commonly used in Cr-induced toxicity studies. Therefore, the study proposes a high risk of brain-targeting injury among individuals environmentally or occupationally exposed to Cr dust, even in low doses, and an additional risk of lung injury with higher Cr concentrations. Therefore, some recommendations must be needed for practitioners of occupational and environmental medicine to be alert for the brain and lung hazards of exposure to Cr dust, and routine physical examinations are needed to assess and inhibit these toxic effects. Besides, further studies are required to find suitable protective and therapeutic agents against these Cr-induced toxicities. Our study suggests neuroprotective agents with anti-inflammatory and anti-oxidant properties as potential candidates.

Moreover, the study introduces inPDC (2 mg/kg)-instillation as the most suitable experimental animal model to study the acute brain and lung toxicities induced by intranasal exposure to Cr. It shows that this model involves the components of oxidative stress and the signaling cascades of inflammation induced by Cr in both organs.
